# Role of TGF-β1 and MAP Kinases in the Antiproliferative Effect of Aspirin in Human Vascular Smooth Muscle Cells

**DOI:** 10.1371/journal.pone.0009800

**Published:** 2010-03-22

**Authors:** Santiago Redondo, Emilio Ruiz, Antonio Gordillo-Moscoso, Jorge Navarro-Dorado, Marta Ramajo, Manuel Carnero, Fernando Reguillo, Enrique Rodriguez, Teresa Tejerina

**Affiliations:** 1 Department of Pharmacology, School of Medicine, Universidad Complutense, Madrid, Spain; 2 Servicio de Cirugía Cardiaca, Hospital Clinico Universitario San Carlos, Madrid, Spain; University of Giessen Lung Center, Germany

## Abstract

**Background:**

We aimed to test the antiproliferative effect of acetylsalicylic acid (ASA) on vascular smooth muscle cells (VSMC) from bypass surgery patients and the role of transforming growth factor beta 1 (TGF-β1).

**Methodology/Principal Findings:**

VSMC were isolated from remaining internal mammary artery from patients who underwent bypass surgery. Cell proliferation and DNA fragmentation were assessed by ELISA. Protein expression was assessed by Western blot. ASA inhibited BrdU incorporation at 2 mM. Anti-TGF-β1 was able to reverse this effect. ASA (2 mM) induced TGF-β1 secretion; however it was unable to induce Smad activation. ASA increased p38^MAPK^ phosphorylation in a TGF-β1-independent manner. Anti-CD105 (endoglin) was unable to reverse the antiproliferative effect of ASA. Pre-surgical serum levels of TGF-β1 in patients who took at antiplatelet doses ASA were assessed by ELISA and remained unchanged.

**Conclusions/Significance:**

*In vitro* antiproliferative effects of aspirin (at antiinflammatory concentration) on human VSMC obtained from bypass patients are mediated by TGF-β1 and p38^MAPK^. Pre-surgical serum levels of TGF- β1 from bypass patients who took aspirin at antiplatelet doses did not change.

## Introduction

Aspirin (Acetylsalicylic acid, ASA) exerts a significant improvement of cardiovascular outcome [1], [2]. Cardiovascular benefits attributed to aspirin have been linked to its antiplatelet effect. Nevertheless, some reports describe the direct antiproliferative effect of ASA on cultured vascular smooth muscle cells (VSMC) [3], [4], which may be related to the decreased carotid atherosclerotic size in patients under ASA treatment [5]. This antiproliferative effect has been linked to the cytokine Transforming Growth Factor-Beta (TGF-β) in rat VSMC [4]. However, no data are available about its link to the effect of ASA on VSMC from patients undergoing coronary artery bypass grafting. The association of ASA and TGF-β in human cardiovascular disease seems plausible, since decreased levels of this cytokine are found in patients suffering unstable angina and they can be restored by ASA treatment [6]. ASA has been shown to decrease plasmatic concentrations of proinflammatory cytokines (IL-6 and CRP) in this clinical condition [7].

The effects of TGF-β are complex and context-dependent. In VSMC, its canonical pathway involves signalling by ALK-5 receptor, which phosphorylates the Smad family proteins and translocate into the nucleus to regulate gene expression [8]. The ALK-5/phospho-Smad2 (PSmad2)-dependent signalling pathway has been linked to apoptosis in human VSMC [9]. Nevertheless, in cultured VSMC the importance of TGF-β1-dependent p38^MAPK^ signalling when TGF-β1 induces proliferation arrest in the absence of apoptosis has equally been described [10]. Interestingly, this antiproliferative but not apoptotic effect of ASA is observed when both rat [4] and human [3] VSMC are incubated with millimolar concentrations of ASA. ASA has been proven to stimulate the p38^MAPK^ pathway in a wide variety of cell types and this effect may be related to its antiproliferative effect [11]. We aimed to demonstrate the effects of both TGF-β1 and the p38^MAPK^ on the antiproliferative effect of ASA in human VSMC, and the effect of clinical consumption of ASA in pre-operative serum levels of TGF-β1.

## Results

### ASA decreases cell proliferation

**Figure 1:**
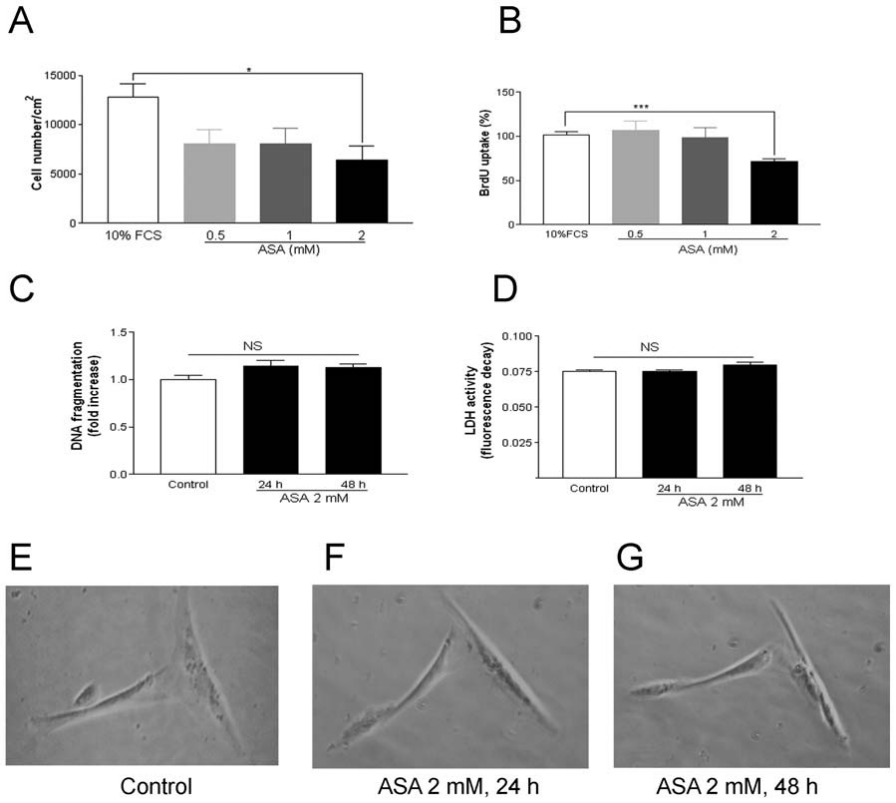
Effects of ASA on human vascular smooth muscle cell proliferation and death. Panel A: Cells were incubated with ASA at 0.5, 1 or 2 mM and counted. Panel B: Concentration-dependency of ASA effect on DNA synthesis. Panel C: Effect of ASA (2 mM) on DNA fragmentation. Panel D: Effect of ASA (2 mM) on LDH activity. Panel E: Photomicrograph shows VSMC cultured in ASA-free10% FCS medium. Panel F: Effect of ASA (2 mM, 24 h) on cell morphology. Panel G: Effect of ASA (2 mM, 48 h) on cell morphology. Bar graphs show the mean±S.E.M of n = 4 experiments (cell cultures from 4 different patients). *P<0.05, ***P<0.001.

ASA decreased cell number at the 2 mM concentration (Figure 1, panel A). Moreover, an ASA-mediated decrease of BrdU uptake was noted (Figure 1, panel B) for the concentration of 2 mM. The highest concentration of the drug proved unable to increase apoptosis (Figure 1, panel C). This lack of effect was also observed when LDH activity was assessed (Figure 1, panel D). In addition, when the cells were observed, they did not show any morphological signs of apoptosis or necrosis after incubation with 2 mM ASA for 24 and 48 h (Figure 1, panels E, F and G).

### Role of TGF-β1

**Figure 2:**
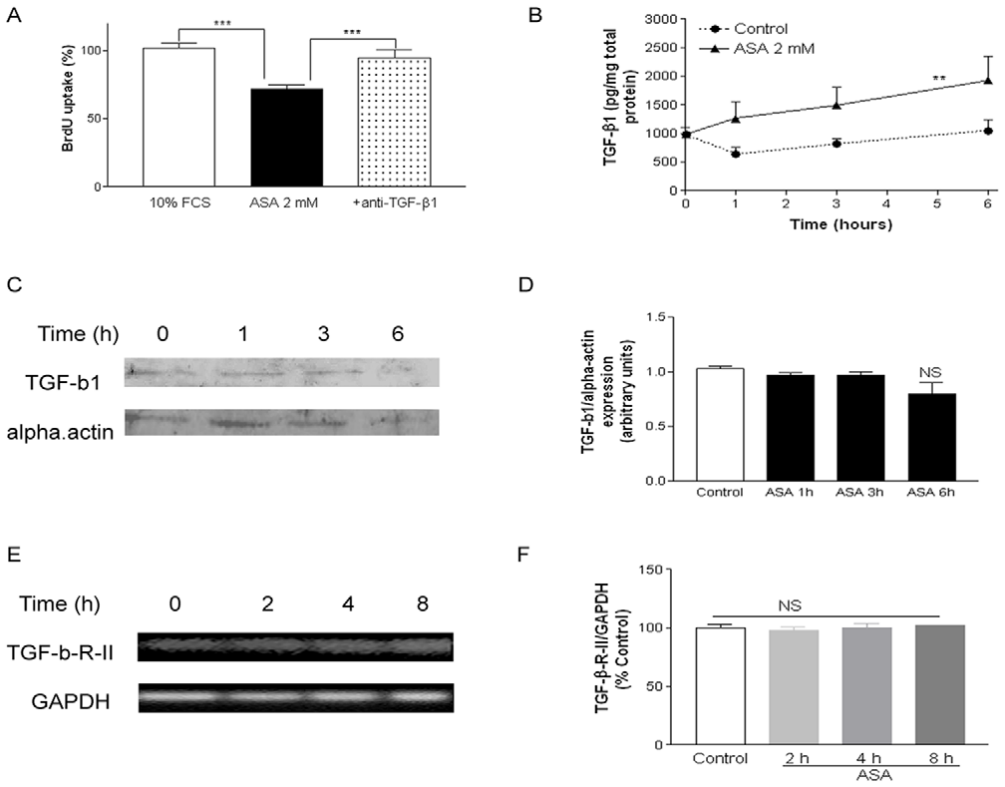
Role of TGF-β1 in the antiproliferative effect of ASA. Panel A: Anti-TGF-β1 (50 µg/ml) reversed the antiproliferative effect of ASA (2 mM). Panel B: ASA-mediated secretion of TGF-β1. Panel C: representative Western blot expression of TGF-β1 after incubation with ASA 2 mM. Panel D: quantification of Western blot experiments. Panel E: Representative RT-PCR blots for TGF-β-RII mRNA after incubation with ASA 2 mM. Panel F: Quantification of RT-PCR experiments. Bar graphs show the mean±S.E.M of n = 4 experiments (cell cultures from 4 different patients). **P<0.01, ***P<0.001.

Inhibition of DNA synthesis induced by ASA (2 mM) was reversed by co-incubation with anti-TGF-β1 antibody (Figure 2, panel A). To assess TGF-β1 secretion and thus reinforce TGF-β1 dependence, ELISA from cell conditioned media with ASA (2 mM) was performed. Figure 2, panel B shows that ASA induces TGF-β1 secretion compared to the control group, where levels initially drop due to the change from serum-rich medium to serum-free medium and subsequently increase due to cell culture secretion (0-6 h). However, ASA (2 mM) was unable to induce protein expression of TGF-β1 in a significant manner (Figure 2, panels C and D). ASA (2 mM) was equally unable to increase TGF-β-R-II transcription (Figure 2, panels E and F).

In ELISA experiments, incubation with ASA 2 mM yields a raw TGF-β1 concentration of 100 pg/ml. When VSMC were cultured with this concentration of exogenously added TGF-β1, it mimicked the effect of ASA 2 mM, since it decreased BrdU incorporation at 19 h (99.78±6.889 vs 68.67±78.99, *P*<0.01). Of note, this concentration of TGF-β1 was equally unable to trigger apoptosis at 24 h (1.0±0.08 vs 0.850±0.080, *P*>0.05).

### ASA does not induce the ALK-5/PSmad2 pathway

**Figure 3:**
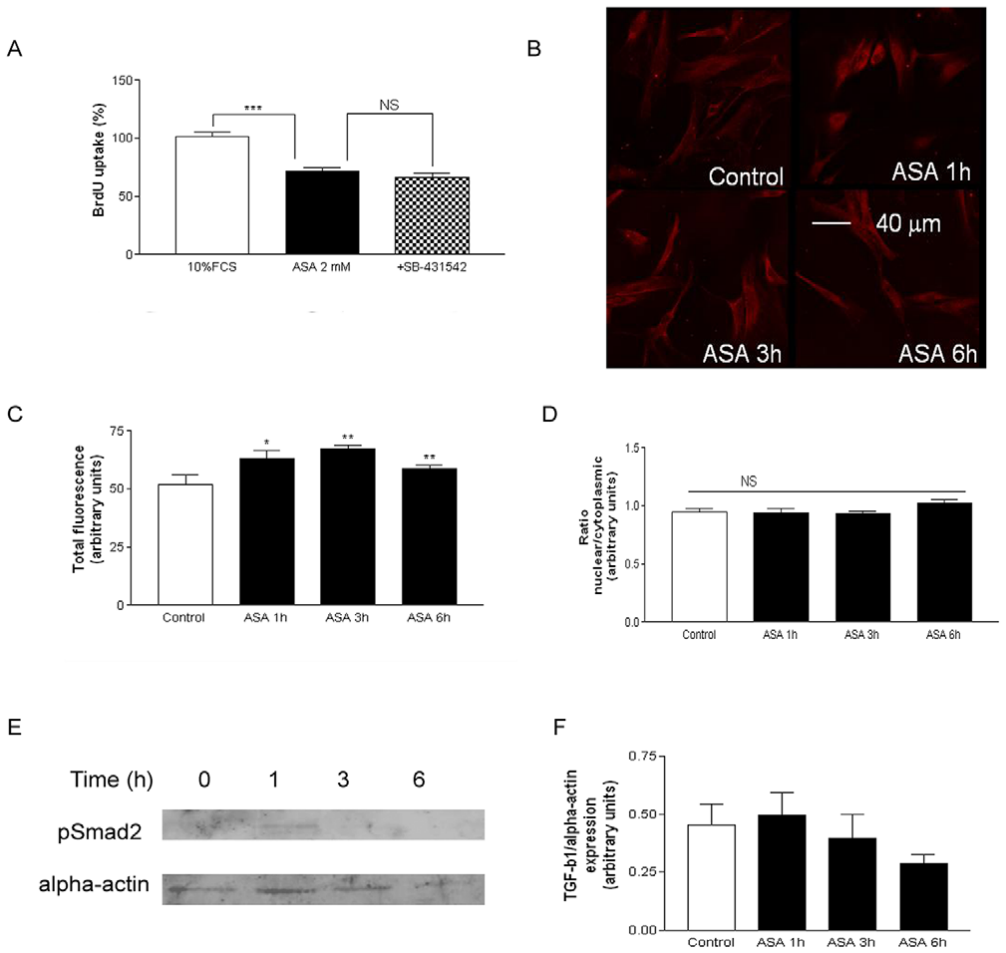
ALK-Smad pathways do not mediate the antiproliferative effect of ASA. Panel A: The ALK-5 blocker SB-431542 (10 µM) did not affect the antiproliferative effect of ASA (2 mM). Panel B: Representative images from cells treated with ASA (2 mM). Panel C: Quantitative analysis of whole-cell intensity of fluorescence in treated *versus* untreated samples. Panel D: Quantitative analysis of nuclear/cytoplasmic intensity in both groups. Panel E: representative Western blots of PSmad2 expression after incubation with ASA (2 mM). Panel F: Quantification of Western blot experiments of PSmad2 after incubation with ASA (2 mM). Bar graphs show the mean±S.E.M of n = 4 experiments (cell cultures from 4 different patients). *P<0.01, **P<0.01, ***P<0.001.

Since the ALK-5/PSmad2 pathway has been involved in the decrease of the proliferation/apoptosis ratio induced by TGF-β1 [9], the antiproliferative effect of ASA was also studied in the presence of the ALK-5 inhibitor SB-431542 (10 µM). The effect of ASA was not inhibited when cells were treated with SB-431542 (Figure 3, panel A). Subcellular location of phosphorylated Smad2 (PSmad2) protein was assessed by confocal microscopy (Figure 3, panel B). ASA enhanced total PSmad2 staining (Figure 3, panel C). However, ASA proved unable to affect its nuclear recruitment. (Figure 3, panel D). In order to rule out Smad2 activation, PSmad2 expression was assessed by Western blot. As shown in Figure 3, panels E and F, basal expression of PSmad2 was very low and ASA (2 mM) was unable to increase it in a significant manner.

### ASA induces TGF-β1-independent p38^MAPK^ phosphorylation

**Figure 4:**
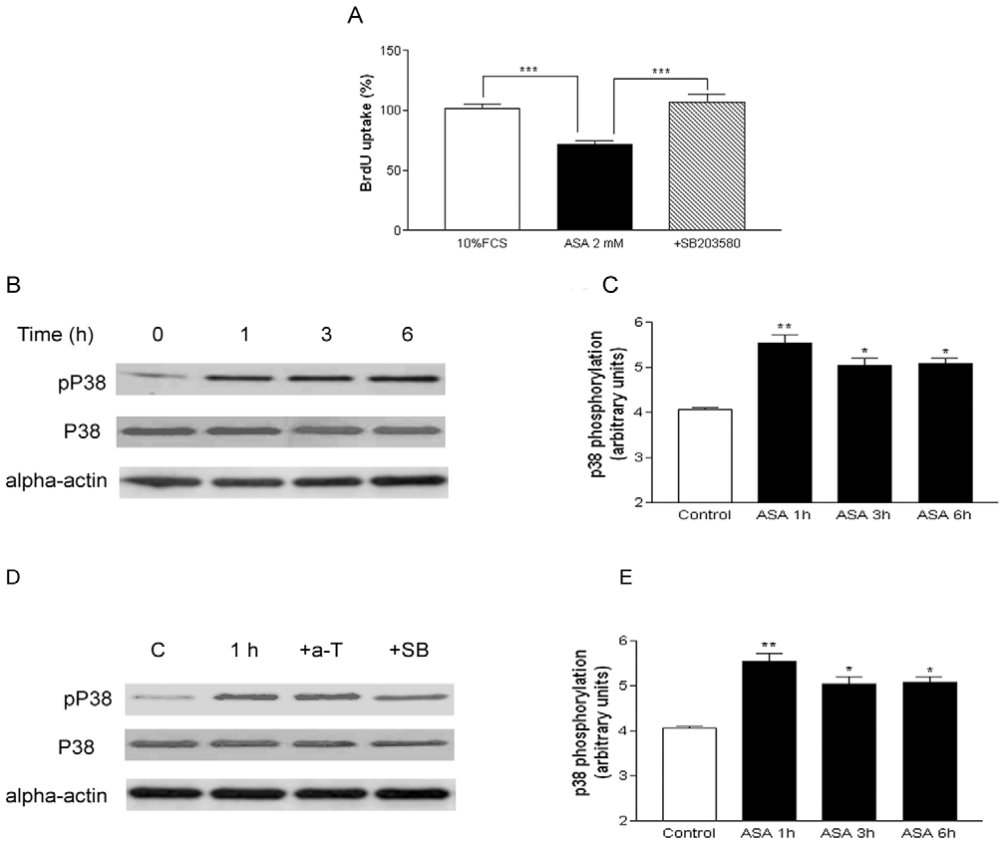
The p38^MAPK^ pathway is involved in the antiproliferative effect of ASA. Panel A: The p38^MAPK^ inhibitor SB203580 (10 µM) reversed the antiproliferative effect of ASA (2 mM). Panel B: Representative blots of p38^MAPK^ phosphorylation after ASA incubation (2 mM) at several time-points. Panel C: quantification of pooled Western blot experiments. Panel D: Representative blots show the role of the TGF-β pathway inhibitors anti-TGF-β1 antibody (50 µg/ml) and SB-431542 (10 µM) on ASA-induced p38^MAPK^ phosphorylation, Panel E: quantification of pooled Western blot experiments. a-T =  anti-TGF-β1 antibody, SB =  SB-431542. Bar graphs show the mean±S.E.M of n = 4 experiments (cell cultures from 4 different patients). *P<0.05, **P<0.01, ***P<0.001.

The p38^MAPK^ inhibitor SB203580 (10 µM) abolished the antiproliferative effect of ASA (Figure 4, panel A). We then tried to assess whether ASA exerts its antiproliferative effect by involving and autocrine loop of TGF-β1 and subsequent p38^MAPK^ activation. Thus, p38^MAPK^ phosphorylation was assessed at 1, 3 and 6 h time, when TGF-β1 was secreted (Figure 2, panel B). When the cells were incubated with ASA, an increase of p38^MAPK^ phosphorylation was observed, reaching a maximum at 1 h (Figure 4, panels B and C). However, neither anti-TGF-β1 (50 µg/ml) nor the ALK-5 inhibitor SB-431542 (10 µM) had a significant effect at 1 h (Figure 4, panels D and E).

### ERK1/2^MAPK^ is not involved in the antiproliferative effect of ASA

**Figure 5:**
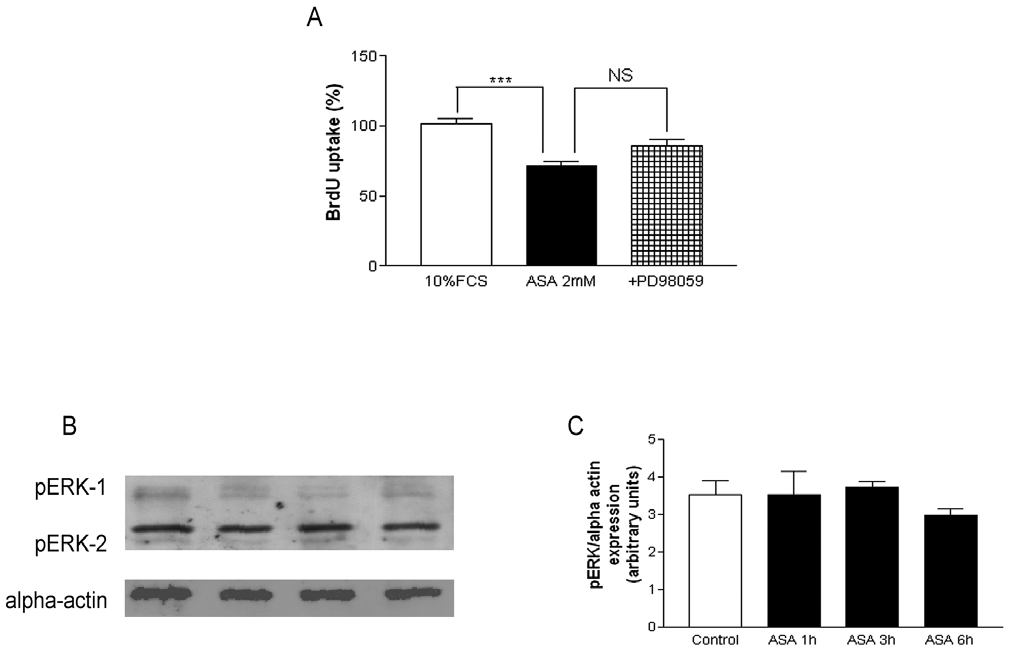
The ERK^MAPK^ pathway is not involved in the antiproliferative effect of ASA. Panel A: Effect of the ERK^MAPK^ blocker PD98059 when added to ASA (2 mM). DNA synthesis was assessed by BrdU uptake, as stated in Methods. Panel B: Representative blots of ERK^MAPK^ phosphorylation (2 mM). Panel C: Quantification of pooled results. Bar graphs show the mean±S.E.M of n = 4 experiments (cell cultures from 4 different patients). ***P<0.001.

We measured the effect of aspirin in ERK1/2^MAPK^ phosphorylation and its relation to the ASA-mediated antiproliferative effect. As shown in Figure 5, panel A, the ERK1/2^MAPK^ inhibitor PD98059 at 10 µM did not reverse the antiproliferative effect of aspirin at 2 mM. Incubation with aspirin did not induce a higher phospho-ERK1/2^MAPK^ expression (Figure 5, panels B and C).

### CD105 is not involved in the antiproliferative effect of ASA

**Figure 6:**
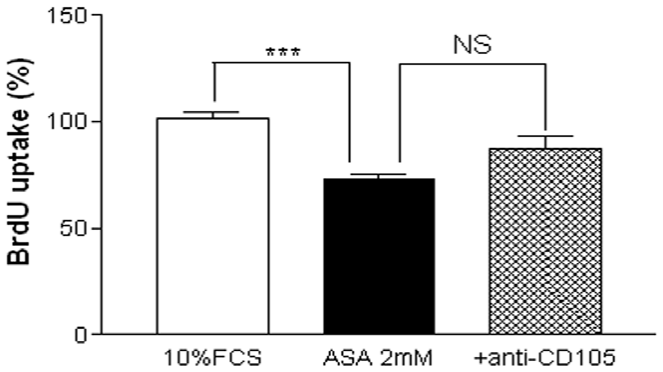
The antiproliferative effect of ASA is independent of endoglin. Effect of anti-CD105 (anti-endoglin, 1 µg/ml) on the antiproliferative effect of ASA (2 mM). Bar graphs show the mean±S.E.M of n = 4 experiments (cell cultures from 4 different patients). ***P<0.001.

Since CD105 or endoglin is an important co-receptor of the TGF-β1 receptor system, we assessed whether CD105 was involved in the antiproliferative effect of ASA. As shown in the Figure 6, coincubation with anti-CD105 (1 µg/ml) was unable to reverse the antiproliferative effect of the drug.

### Therapeutics with aspirin does not increase serum levels of TGF-β1

As shown in the Table 1, when bypass patients were given aspirin at antiplatelet doses (75–300 mg), concentration of TGF-β1 in the pre-surgical serum remained unchanged. Clinical and biochemical characteristics of the patients are shown in Table 1. Our patients showed a high presence of cardiovascular risk factors, in accordance to advanced atherosclerotic disease.

**Table 1:** Clinical characteristics of the study groups.

	No Aspirin n = 57	Aspirin takers n = 64	P
Age (years)	64.4±9.2	64.6±8.8	0.88 *
Female (%)	9 (7)	8 (6.6)	0.60 ‡
Hypertension (%)	29 (24)	38 (31)	0.35 ‡
Diabetes Mellitus (%)	18 (15)	36 (29)	0.006 ‡
Current smokers (%)	23 (19)	15 (12)	0.045‡
Statin takers (%)	22 (18)	42 (35)	0.002‡
Total Cholesterol (mg/dl) ^¥^	161.6±53	146.3±38	0.05 *
LDL-cholesterol (mg/dl) ^¥^	88±41	77±32	0.09 *
HDL-cholesterol (mg/dl)	37.5±13.5	38.5±10.25	0.78§
Triglycerides (mg/dl) ^¥^	147.6±68	128.2±64	0.05 *
C- reactive Protein (mg/l)	4.3±8	3.6±5	0.39 §
TNF-α (pg/dl)	6.3±2.7	6.0±3.2	0.39 §
TGF-β1 (ng/ml)	43±14.4	39±12.3	0.11 *
TGF-β1/No.platelet ^¥^	0.17±0.8	0.17±0.6	0.99 *

Data represent the mean±S.D (^¥^ Harmonic mean). Data with non-parametric distribution (HDL-cholesterol, C-reactive protein and TNF-α) are shown as median±IQR.

*Student t test.

‡ Squared-chi test.

§ Mann-Whitney U test.

Significance is assumed for a *P<*0.05.

## Discussion

The most relevant findings of this work can be summarized as follows: 1) At milimolar concentrations ASA has an antiproliferative effect on human VSMC from bypass patients; 2) This antiproliferative effect is mediated by the release of TGF-β1; 3) The intracellular mechanism of ASA does not involve the activation of Smad2 but the activation of p38^MAPK^ independently of the TGF-β1; 4) The early activation of TGF-β1 and p38^MAPK^ are able to trigger a maintained antiproliferative effect at 24 h; 5) Serum levels of TGF-β1 are not changed in bypass patients who take aspirin at antiplatelet doses. Thus, the present study is the first report which demonstrates the role of TGF-β1 and MAP kinases in the antiproliferative of ASA on human VSMC. Moreover, these VSMC were obtained from severe atherosclerotic patients ongoing bypass surgery, which highlights the potential importance of these molecular mechanisms in samples obtained from the clinic.

An important finding of the present work is the inability of the ALK-5 inhibitor SB-431542 to reverse the antiproliferative effect of ASA, in contrast to the observed reversion by anti-TGF-β1. This lack of effect of SB-431542 is accompanied by the failure of ASA to induce PSmad2 expression in our cell model. Interestingly, ALK-5 and Smad2-dependent pathways have been described to mediate TGF-β1-induced apoptosis of rat [12] and human VSMC [9], which coincides with the inability of ASA to trigger cell death (Figure 1, panels C and D). At the same time, endoglin or CD105 is an accessory receptor of the TGF-β1 which has been closely related to the ALK/Smad receptor system [8]. In our experiments, anti-CD105 was unable to reverse the antiproliferative effect of ASA (Figure 6). Thus, as a whole, our data suggest that the ASA-inducedTGF-β1 signalling does not involve the classical ALK/Smad system. Non-Smad pathways are emerging TGF-β1 signalling mechanisms which seem to play an important role in VSMC cycle control [8]. The antiproliferative but not apoptotic effect of ASA on human VSMC described in the present study reproduces what reported elsewhere [3].

In the present report we find an ASA-mediated TGF-β1 increase in the cell culture conditioned media (Figure 2, panel B). In a previous study, however, we found that aspirin was unable to increase TGF-β1 secretion in a rat VSMC [4]. Inter-species differences, in addition to the advanced age and atherosclerotic evolution of our group of patients may mediate this difference. COX-1, the major target of ASA, regulates exocytosis of platelet granules and it might mediate VSMC secretion of TGF-β1 after incubation with the drug [3].

Given that ALK-5/Smad2 pathway was not involved in the antiproliferative effect of ASA we looked at another intracellular pathway related with TGF-β1. The p38^MAPK^ inhibitor SB203580 significantly reduced the effect of ASA (Figure 4, panel A). However, the activation of p38^MAPK^ was independent of TGF-β1 since neither the anti-TGF-β1 antibody nor the ALK-5 inhibitor was able to block the effect of the drug (Figure 4, panel D and E). These data confirm that the antiproliferative effect of ASA was mediated by two independent mechanisms: a TGF-β1-dependent (Figure 2, panel A) and a p38^MAPK^-dependent mechanism (Figure 4, panel A). This last mechanism involving p38^MAPK^ may be mediated by the salicylic part of the molecule [3]. On the other hand, aspirin-mediated increase in basal p38^MAPK^ and decrease of other inflammation-related proteins such as ERK1/2^MAPK^ has been reported [11], [13]. This study suggests a non-significant effect of basal ERK1/2^MAPK^ in the antiproliferative role of aspirin (Figure 5, panels A, B and C).

The range of *in vitro* concentrations of ASA used in this study was related to antiinflammatory doses [11]. Cardiovascular benefits attributed to ASA are generally explained by its antiplatelet action, due to its irreversible inhibition of platelet COX-1 by irreversible acetylation of Ser 530 [14]. Nevertheless, *in vitro* concentrations of the drug in cell culture media and clinical doses of the drug cannot be correlated on an exact basis, since cell culture models lack several pharmacokinetic phenomena such as accumulation, bio-disposability and the existence of different pharmacokinetics compartments. Normal doses of ASA for cardiovascular purposes (such as 300 mg) are up to 10 times over the optimal concentration of COX-1 inhibition [11]. Although there is a proven *in vitro* antiplatelet activity in patients under ASA treatment [15] the idea of additional mechanisms for ASA-induced cardiovascular protection has been strengthened by some findings about its antiinflammatory and antiproliferative effects [3], [4], [11].

Benefits of CABG surgery are still dampened by its rate of graft failure, which may be ameliorated by means of ASA therapy [16]. In-vessel graft failure determinants include endothelial damage, thrombosis and VSMC growth [17]. The question arises whether *in vitro* millimolar concentration relate to the ones achievable *in vivo* by using clinical doses of the drug. At antiinflammatory doses, ASA concentration in plasma reaches from 0.95–1.9 mM, with a concentration of free drug of 250 µM. Since incubation media contains 10% serum, millimolar *in vitro* concentration has been regarded as related to anti-inflammatory dose [11], although the concept that drug concentrations in cell culture media mimic the ones obtained from clinical plasma cannot be demonstrated.

Nevertheless, it is clear that high anti-inflammatory doses of aspirin may produce an increase of undesirable effects. On the other hand, the at least equal efficacy of low-dose aspirin (75–150 mg) compared to higher ones for long-term cardiovascular treatment is sustained by strong evidence [2]. Moreover, a controlled clinical trial to compare aspirin at 50 and 900 mg did not find any difference of re-occlusion after aortoiliac and femoropopliteal percutaneous transluminal angioplasty, whereas the high dose group had a higher rate of gastrointestinal side effects [18].

Platelets play an essential role in atherosclerosis not only from a haemostatic point of view but also by acting as direct inflammatory mediators [19]. A potential way to enhance the therapeutical benefit of ASA at the same time high dose-related adverse effects are avoided may be based on the synergistic use of other antiplatelet agents such as clopidrogrel. A recent large multicentric trial did not observe a significant advance of combined treatment for cardiovascular prevention in atherothrombotic or high-risk patients [20]. However, evidence suggests a significant clinical improvement in myocardial infarct patients treated with both drugs [21]. Combination therapy seems to exert a significant benefit in bypass patients [22]. Whether these synergistic clinical benefits take place, at least in part, through antiinflammatory cytokine pathways and the role of platelets when this effect is considered will be the subject future research.

In conclusion, in the present study we show that at *in vitro* antiinflammatory concentrations ASA exerts a TGF-β1and p38^MAPK^-dependent antiproliferative effect on human VSMC. At the same time, however, aspirin therapy at antiplatelet doses does not change the serum levels of TGF-β1 in our clinical sample. Translational strategies in order to extend the *in vitro* additional benefits of aspirin to the clinical scenario will be the subject of further research.

## Materials and Methods

### Ethics Statement

Clinical data were obtained from bypass surgery patients at the Cardiac Surgery Service (Hospital Clinico San Carlos, Madrid, Spain). Exclusion criteria were age≥80, inflammatory co-morbidities, end-stage renal disease and cancer. The study was conducted according to the Declaration of Helsinki. The study was approved by the Local Ethical Committee (Hospital Clinico San Carlos, Universidad Complutense, Madrid, Spain). In addition, patients gave written informed consent. The internal mammary artery is the vessel of choice for coronary artery bypass grafting, since this vessel is free from atherosclerotic lesions. Experiments were only made with remaining samples after surgery.

### Cell cultures

VSMC from internal mammary arteries obtained from our clinical sample were cultured from explants in RPMI (Life Technologies, Barcelona, Spain) containing 10% foetal calf serum (FCS). Cells exhibited typical “hill and valley” morphology and were stained with a monoclonal anti-smooth α-actin. Cells at passages 3–5 were used (4000 cells/cm^2^). VSMC were obtained from the internal mammary arteries following the explant technique. Cell cultures took a minimum of 1–2 months before going viable. During all that time, VSMC were incubated in aspirin-free medium.

### Cell counting

Cells were seeded onto 24-well plates and allowed to attach for 24 h. They were then treated with ASA at 0.5, 1 or 2 mM. After 24 h, they were detached with trypsin and counted on a Neubauer's chamber.

### LDH assay

Lactate dehydrogenase (LDH) activity was assessed as a measurement of cytotoxicity according to a method previously described [23]. Briefly, cells were incubated in the presence or absence of ASA at 2 mM. After 24 h lactate-dehydrogenase (LDH) activity was measured in the supernatant by pyruvate decay at 340 nm.

### DNA fragmentation

Cellular DNA fragmentation was measured with a commercial ELISA kit (Roche-Boerhinger, Spain). Proliferating cells in 96-well plates were labelled with 10 µM BrdU overnight and treated with ASA. They were incubated with the kit lysis buffer (BSA, EDTA, and Tween® 20) for 30 min at room temperature. Soluble BrdU-labelled DNA fragments were quantified using the ELISA kit.

### Cell morphology

VSMC were plated on F25 cell culture flasks (Nunc, Roskilde, Denmark), and allowed to attach overnight. They were then incubated with ASA-free 10% FCS medium or with ASA 2 mM for 24 or 48 h. Images were obtained using a DMT4000-B inverted microscope (Leica, Wetzlard, Germany) and registered by a FE-3000 digital camera (Olympus, Tokyo, Japan).

### BrdU incorporation

VSMC were plated onto 96-well plates and allowed to attach for 24 h. The cells were serum-starved for 32 h in 0.4% FCS containing medium in order to synchronize the cells in G_0_/G_1_. They were then treated with 10% FCS-containing medium with ASA (0.5, 1 or 2 mM) for 19 h, and loaded with BrdU (10 µM) for the last 3 h of treatment. BrdU incorporation was measured by an ELISA kit (Amersham Life Science, Barcelona, Spain). Inhibitors were: anti-TGF-β1 at 50 µg/ml (R&D Systems, Minneapolis, MN), the p38^MAPK^ inhibitor SB203580 (10 µM), the ERK^MAPK^ inhibitor PD98059 (10 µM), the ALK-5 blocker at 10 µM SB-431542 (Tocris, Bristol, UK), and anti-CD105 at 1 µg/ml (BD Bioscience, Franklin Lakes, USA), as described [24].

### ELISA measurements from cell conditioned media

Total TGF- β1 levels were determined by a TGF-β1-specific ELISA, (R&D Systems, Minneapolis, MN). Cells were incubated with ASA 2 mM in serum-free medium (RPMI+ 0.2% bovine serum albumin + PDGF-BB at 10 ng/ml).

### Reverse transcription PCR (RT-PCR)

Sensitivity of VSMC cultures to exogenous TGF-β1 is strongly associated with TGF-β-R-II expression [25]. We aimed to assess this parameter by RT-PCR. Total RNA was extracted using a commercial kit with DNase (RNeasy Mini kit, Qiagen, Spain). RT-PCR was performed using the Titan-one-tube RT-PCR (Boehringer, Mannheim, Germany). cDNA was obtained by reverse transcription at 50°C (30 min) and 95°C (5 min). The TGF-β-R-II gene was amplified using specific conditions, as described [26]. Oligonucleotides are listed below:

TGF-β-R-II forward primer: CTACAAGGCCAAGCTGAAGC

TGF-β-R-II reverse primer: AGCCATGGAGTAGACATCCG

GAPDH forward primer: CGATGCTGGCGCTGAGTA

GAPDH reverse primer: CGTTCAGCTCAGGGATGACC

Thermal profile used a BioRad thermal cycler for denaturation at 95°C for 1 min, annealing at 55°C for 1 min, and extension at 72°C for 1 min (23 cycles).

### Confocal microscopy

Cells were cultured in the presence or absence of ASA 2 mM from 1 to 6 h. The cells were washed with PBS, fixed for 20 min in 4% paraformaldehyde in PBS and permeabilized with 0.4% triton-x100 for 30 min at room temperature. After blocking with 3% BSA in PBS, the cells were then incubated with rabbit polyclonal anti-PSmad2 (Calbiochem, Schwabach, Germany), at 1∶100 for 1 h, following by incubation with goat anti-rabbit Alexa 568® (1∶100, Molecular, Probes, 1 h). Images were captured using a Leica-TCS-SP2 inverted microscope. Intensity was analyzed by Image J.

### Western blot

Cells were treated with ASA 2 mM in 10% FCS-containing medium (1 to 6 h). They were washed with ice-cold PBS, and lysed on ice with 200 µl lysis buffer (10% glycerol, 2.3% SDS, 62.5 mM Tris, pH 6.8 150 mM NaCl, 10 mM EDTA, 1 µg/ml eupeptic, 1 µg/ml pepstatin, 5 µg/ml chymostatin, 1 µg/ml aprotinin, 1 mM phenylmethylsulphonyl fluoride) and boiled for 5 min. Equal amounts of protein were run on 10% SDS-polyacrylamide electrophoresis, transferred to polyvinylidene difluoride (PVDF) membranes (Immobilon-P, Amersham, Madrid, Spain), and blocked overnight at 4°C in blocking solution (5% skimmed milk in TBS-T: 25 mM Trizma base, 75 mM NaCl, pH 7.4, 0.1% v/v Tween® 20). Blots were incubated with agitation at 4°C overnight with anti-phospho-p38^MAPK^ or anti-p38^MAPK^ (both of them from Calbiochem, Schwabach, Germany) at 1∶1000 in TBS-T. Blots were incubated for 1 h at room temperature with horseradish peroxidase anti-rabbit-IgG (Santa Cruz Biotechnology, CA, 1∶10000). Protein was visualized with ECL (Amersham Biosciences, Barcelona, Spain).

Similar procedures were used for Western blot expression assessment of TGF-β1 (anti-TGF-β1, Santa Cruz, Biotechnologies, Santa Cruz, USA), phospho-Smad (anti-Smad2, phospho-specific, Calbiochem, Schwabach, Germany) and phospho-ERK (anti-active ERK^MAPK^, Calbiochem, Schwabach, Germany). Smooth muscle α-actin was used as a housekeeping protein (anti-α-actin, Sigma-Aldrich, Madrid, Spain). All the primary antibodies were diluted at 1∶1000 in TBS-T.

### Determination of serum TGF-β1

Pre-surgical serum was taken from the patients who were chronically treated with aspirin at antiplatelet doses (75–300 mg daily, n = 64), or not (n = 57). Total levels of TGF-β1 (active plus acid-activatable) were assessed by ELISA (R&D systems, Minneapolis, MN, USA). This kit proved highly reproducible in a large comparative study of several ELISA kits for TGF-β [27].

### Statistical analysis

A statistical analysis of the data was carried out by a Student's t test, Fisher exact test, or by a one-way ANOVA when necessary.

### Drugs and reagents

Acetylsalicylic acid (ASA) was purchased from sigma (Madrid, Spain), and dissolved in the cell cultured medium. Specific pharmacologic inhibitors (all from Tocris, Bristol, UK) of p38^MAPK^ (SB203580), ERK^MAPK^ (PD98059) and ALK-5 (SB-431542) were preincubated at 10 µM 1 h before the stimulus was added and then they were coincubated, again at 10 µM, for the indicated time points. This strategy has been demonstrated to totally inhibit these pathways [28], [29], [30]. Anti-TGF-β1 (R&D systems, Madrid, Spain) was coincubated at 50 ng/ml, since this approach was able to totally inhibit TGF-β1 in vascular smooth muscle cells [4], [31]. Anti-CD105 (BD Bioscience, Franklin Lakes, USA) was coincubated at 1 µg/ml in order to totally block CD105, as described [24].
